# Supplementary material for: SynBot: An open-source image analysis software for automated quantification of synapses
Source: bioRxiv. 2024 Jul 12:2023.06.26.546578. Originally published 2023 Jun 28. Preprint. [Version 4] doi: 10.1101/2023.06.26.546578 (PMC10327002; doi:10.1101/2023.06.26.546578)
Supplement: Supplement 2 [file media-2.pdf]

Key resources table

REAGENT or RESOURCE	SOURCE	IDENTIFIER
Antibodies		
AffiniPure Goat anti-Rat IgG + IgM (H+L)	Jackson Immunoresearch	Cat# 112005044<br>RRID: AB_2338094
AffiniPure Goat anti-Mouse IgG + IgM (H+L)	Jackson Immunoresearch	Cat# 115005044<br>RRID: AB_2338451
Mouse anti-neural cell adhesion molecule L1 Hybridoma	Developmental Studies Hybridoma Bank	Cat# ASCS4<br>RRID: AB_528349
Anti-Bassoon antibody	Enzo/Assay Designs	Cat# SAP7F07/VAM-PS003F<br>RRID: AB_2038857
Anti-Gephyrin antibody	Synaptic Systems	Cat# 147002<br>RRID: AB_2619838)
Anti-Homer1 antibody	Synaptic Systems	Cat# 160002<br>RRID: AB_2120990
Anti-VGAT antibody	Synaptic Systems	Cat# 131004<br>RRID: AB_887873
Anti-Vglut1 antibody	Millipore	Cat# AB5905<br>RRID: AB_2301751
Alexa Fluor 488 goat anti-Mouse IgG (H+L)	Invitrogen	Cat # A11001<br>RRID: AB_2534069
Alexa Fluor 568 goat anti-Rabbit IgG (H+L)	Invitrogen	Cat# A11011<br>RRID: AB_143157
Alexa Fluor 647 goat anti-Guinea pig IgG (H+L)	Invitrogen	Cat# A21450<br>RRID: AB_2535867
Guinea pig anti- VGAT	Synaptic Systems	Cat# 131004<br>RRID: AB_887873
Rabbit anti-Gephyrin	Synaptic Systems	Cat# 147002<br>RRID: AB_2619838
Rabbit anti-PSD95	Life Technologies	Cat# 51-6900<br>RRID: AB_2533914
Chemicals, peptides, and recombinant proteins		
2,2,2-tribromoethanol	Sigma	Cat# T48402-25G
2-methyl-2-butanol	Sigma	Cat# 152463-250mL
B27	GIBCO	Cat# 17504044
B27 Plus	GIBCO	Cat# A3582801
BDNF	PeproTech	Cat# 450-02
Boric Acid	Sigma	Cat# B0394
BSA	Sigma	Cat# A4161
BSL1 (Baneiraea Simplicifolia Lectin 1)	Vector Laboratories	Cat# L-1100
CNTF	PeproTech	Cat# 450-13
Cytosine arabinoside (AraC)	Sigma	Cat# C1768
DAPI	Invitrogen	Cat#D1306
DMEM	GIBCO	Cat# 11960
DNaseI	Worthington	Cat# LS002007

DPBS with calcium, magnesium, glucose, and pyruvate	GIBCO	Cat# 14287
DPBS without calcium or magnesium	GIBCO	Cat# 14190144
Fetal Bovine Serum	Thermo Fisher	Cat# 10-437-028
Forskolin	Sigma	Cat# F6886
Glycerol	Acros Organics	Cat# 15892-0010
Hydrocortisone	Sigma	Cat# H-0888
Insulin	Sigma	Cat# 11882
L-Glutamine	GIBCO	Cat# 25030-081
Low protein binding tubes	Eppendorf	Cat# 022431081
Mouse Laminin	Cultrex	Cat# 3400-010-01
N-acetyl cysteine	Sigma	Cat# A8199
n-Propyl gallate	Sigma	Cat# P3130-100G
Neurobasal	GIBCO	Cat# 21103049
Neurobasal minus phenol red	GIBCO	Cat# 12348017
Neurobasal Plus	GIBCO	Cat# A3582901
Normal Goat Serum (NGS)	Thermo Fisher	Cat# 01-6201
Optimal Cutting Temperature solution (OCT)	Tissue Tek	Cat# 4583
Papain	Worthington	Cat# LK003178
Pen/Strep	GIBCO	Cat# 15140
PFA 16%	Electron Microscopy Sciences	Cat# 15710
Poly-D-Lysine	Sigma	Cat# P6407
Sodium Pyruvate	GIBCO	Cat# 11360-070
Tris Base	VWR	Cat# 101174-856
Triton X-100	Roche	Cat# 11332481001
Trypsin Inhibitor	Worthington	Cat# LS003083
Critical commercial assays		
Pierce BCA protein assay kit	Thermo Fisher	Cat# 23225
Deposited data		
Synapse microscopy images	This paper	DOI: 10.5281/zenodo.12191805
Experimental models: Cell lines		
Rat primary cortical neurons	This paper	N/A
Rat primary cortical astrocytes	This paper	N/A
Experimental models: Organisms/strains		
Rat: Sprague-Dawley	Charles River	001

Software and algorithms		
SynBot (version 1.1.1)	This paper	Eroglu-Lab/Syn_Bot: Syn_Bot synapse calculation macro for FIJI (github.com)<br><br>https://doi.org/10.5281/zenodo.12192447
FIJI (version 2.14.0)	NIH	https://fiji.sc/<br>RRID:SCR_002285
Ilastik (version 1.3.3)	Anna Kreshuk's lab (EMBL)	https://www.ilastik.org/<br>RRID:SCR_015246
Puncta Analyzer (version 2.0)	Ippolito and Eroglu, 2010	https://github.com/toddstavish/puncta-analyzer
R: A Language and Environment for Statistical Computing (version 4.3.3)	R Core Team	https://cran.r-project.org/<br>RRID:SCR_001905
R package nlme (version 3.1-164)	R Core Team	https://www.rdocumentation.org/packages/nlme/versions/3.1-162<br>RRID:SCR_015655
Other		
20µm nylon mesh	Elko filtering	Cat# 03-20/14
Vivaspin MWCO 5000; 20 mL tubes	Sartorius	Cat # VS2012
